# In-Plane Deformation Behavior and the Open Area of Rotating Squares in an Auxetic Compound Fabric

**DOI:** 10.3390/polym14030571

**Published:** 2022-01-31

**Authors:** Polona Dobnik Dubrovski, Nejc Novak, Matej Borovinšek, Matej Vesenjak, Zoran Ren

**Affiliations:** Mechanical Engineering Research Institute, Faculty of Mechanical Engineering, University of Maribor, 2000 Maribor, Slovenia; nejc.novak@um.si (N.N.); matej.borovinsek@um.si (M.B.); matej.vesenjak@um.si (M.V.); zoran.ren@um.si (Z.R.)

**Keywords:** compound textile material, auxetic structure, open area, filtration, mechanical properties, Poisson’s ratio

## Abstract

A conventional compound fabric was used to develop a modern, multifunctional material with an auxetic behaviour and a tailored open area for particle filtration. Such material was produced using traditional textile technology and laser cutting, to induce a rotating squares unit geometry. The behaviour was investigated of three different rotating unit cell sizes. The laser slit thickness and the length of the hinges were equal for all three-unit cells. The tensile properties, Poisson’s ratio and auxetic behaviour of the tested samples were investigated, especially the influence of longitudinal displacement on the fabric’s open area and the filtered particle sizes (average and maximum). Results show that the developed compound fabric possesses an average negative Poisson’s ratio of up to −1, depending on the applied auxetic geometry. The larger rotating cell size samples offer a higher average negative Poisson’s ratio and a higher breaking strength due to the induced slits. The findings highlight the usefulness of patterned cuts in conventional textile materials to develop advanced auxetic textile materials with tailored geometrical and mechanical properties.

## 1. Introduction

The textile industry is also focused on the development of textiles with a negative Poisson’s ratio [1,2,3,4,5,6,7,8,9,10] at different levels of textile forms, e.g., fiber, yarn and fabric’s levels. The auxetic properties of fibers are a result of their unique structure. In the case of yarns and fabrics, auxeticity can be reached in two ways: (a) With induced auxetic geometry using standard fibers/yarns during the processes of spinning, weaving, knitting, braiding, and nonwoven production, or (b) By using auxetic fibers (and, from them, the produced yarns) within the current above mentioned textile production processes.

Alderson & Evans performed the first laboratory attempt to develop auxetic fibers [1]. They developed a polymer–an ultra-high molecular weight polyethene (UHMWPE), which had a negative Poisson’s ratio (NPR) of −1.2. The polymer had a microporous node-fibril structure that consisted of a network of rectangular nodes (surface-melted powder particles) connected by freely hinged inextensible rods/fibrils. Such an arrangement was achieved by a three-stage thermal processing route with extrusion of the sintered UHMWPE rod through a die as the final stage [2]. Extrudes were created in cylindrical rods with a diameter between 8 mm and 15 mm [3]. Then, Alderson et al. made auxetic PE fibers [4] and auxetic polypropylene (PP) fibers successfully, based on a modified conventional melt spinning technique on an industrial scale [5]. Ravirala et al. produced auxetic polyester (PES) [3,6] and polyamide (PA) [6] fibers by a method that originated from a technique used for creating auxetic PP fibers. He et al. [7] created auxetic liquid crystalline polymers (polyether and polyester). The polymer main chain had laterally attached (para-quaterphenyl) rods, which could rotate to up to 75° in the case when the polymer was stretched, thus exhibiting an auxetic behavior.

Auxetic yarns with induced auxeticity are specially constructed plied yarns with two types of conventional single yarns of different fineness and stiffness. The helical auxetic yarns (HAY), invented by Hook [8], contain a core yarn and a wrap yarn. The core yarn is thick, while, around the core yarn, the helically wound warp yarn is finer and stiffer. Under tension, the finer wrap yarn starts to uncurl, while the thicker core yarn starts to move laterally, thus increasing the yarn’s width and achieving a negative Poisson’s ratio. Wright et al. tested different HAY yarns made from a combination of core yarn (rubber yarns with diameters of 0.18 mm, 1 mm and 2 mm) and wrap yarn (textured PA multifilament yarn of 110 tex, and polyethene terephthalate (PET) ring-spun yarn of 63 tex) with 45° of nominal angle [9]. Sloan et al. [10] performed an experimental study of the auxetic properties of simple HAYs made from polyurethane monofilament core yarn (with 600 µm in diameter) and PA monofilament warp yarn (with 110 µm, 130 µm, and 150 µm in diameter). The maximal auxetic effect of the tested HAYs was −2.7. Ge et al. [11] developed HAY with a 4-ply structure, and Ng et al. [12,13] with 4- and 6-ply structures. When such yarn is stretched longitudinally, the stiff yarns tend to move to the yarn center and push the less stiff yarns out of the yarn center, thus resulting in the lateral extension of the auxetic yarn.

Another way to develop low-cost auxetic fabrics is by inducing a unique fabric structure with auxetic geometry in the traditional textile processes using conventional yarns or fibers. The re-entrant geometry [14,15,16,17,18] and foldable geometry [14,19,20,21] have been used to develop novel woven and knitted fabrics with in-plane auxetic behavior. Rotating unit geometry was used by Hu et al. [14] to create auxetic weft-knitted fabrics with rotating rectangles. Such structure was achieved by partial interlock knitting to form rectangle units, and by the binding-off technique with elastic yarn to form a connection of rectangles in the wale direction. Zulifqar et al. [22] developed auxetic woven fabrics by inducing rotating rectangle geometry. The unit cell consisted of “four rigid rectangles connected at their vertices by hinges” [22], and had rhombi-shaped empty spaces between the rectangles. Such a structure involved three different areas regarding the type of weave, e.g., a tightly woven area (rectangle unit), a loosely woven area and an interlacement free area. The fabrics were made from non-elastic warp yarns and alternately elastic and non-elastic cotton weft yarns. Rotating unit geometry was also used in developing auxetic nonwoven fabrics. Sharma et al. [23] designed and developed needle-punched polypropylene webs. A special structure was achieved with slit perforations to form different rotating units (squares, quadrilaterals). Such structures induced in-plane auxetic behavior of the nonwoven fabrics. Bhullar et al. [24,25] also used the rotating square geometry in polycaprolactone nanofiber webs (membranes) made by the electrospinning procedure and laser cutting. Rawal et al. [24] developed 3D polyester needle-punched webs with out-of-plane auxetic behavior by adjusting the needle boards‘ direction in a needle-punching process (and, consequently, the proportion of fibers in the through-thickness direction of the webs). Rawal et al. [25] analyzed needle-punched nonwoven fabrics with out-of-plane auxetic behavior by deconstructing the anisotropic structure, and presented analytical models of the modulus and an in-plane Poisson’s ratio. Verma et al. [26] developed 3D polyester needle-punched webs with out-of-plane auxetic behavior, based on the heat compression protocol after fabrication of a needle-punched fabric. They showed that “during the heat compression treatment, fiber bundles occupied a buckle/tilted state, which opened up during mechanical extension and, hence, web thickness increased and out-of plane auxetic effect was achieved” [26].

Grima et al. [27,28,29] first proposed the rotating rigid unit cell geometry by joining the rigid units (triangles, squares, rectangles) through hinges at their corners. Grima et al. [30,31,32] further analyzed various types of networks based on rotating rigid units (rhombi and parallelograms).

In our previous research [33], the auxetic behavior of modified conventional non-woven fabrics based on the rotating rigid unit cell geometry was investigated. We found that non-woven samples with smaller rotating cell sizes exhibited the highest negative Poisson’s ratio. The presented research also dealing with auxetic behavior of fabrics, but it is focused on another textile structure, e.g., compound fabric, and on the open area during the tensile loading as the consequence of cells’ rotation. A brief overview of existing types of auxetic textile structures shows that no one has developed an auxetic compound fabric yet, nor tried to transform the conventional compound fabric into an auxetic one. Compound fabrics have a more complex structure and different behavior under tensile force than non-compound fabrics such as woven, nonwoven and knitted fabrics. 

Although the geometry of rotating cells has been investigated widely, it has been studied less on a textile substrate, especially on compound fabric. The findings of the presented research disseminate knowledge about the behavior of auxetic compound fabric under quasi-static loadings. The geometry of rotating cells was used to fabricate the auxetic compound fabric used as a transportation and filtration material in this research. While the conventional filters are designed only for one-size particle filtration, the goal of the research was also to develop a novel filtration material with an adjustable open area, to have the possibility to change the opening size for filtration of different sized particles. The latter can be achieved based on the adequate rotating cell geometry induced into the fabric, and sufficient tensile force applied to the fabric, needed for cells’ rotation. 

Conventional compound fabric, e.g., needle-punched nonwoven fabric reinforced with woven fabric, was used as the basis, which was then laser cut to form the rotating squares structures and, thus, auxetic compound fabrics. In addition, in-plane deformation behavior under quasi-static-tensile load of conventional and auxetic compound fabrics, the Poisson’s ratio, and the relationship between the fabric’s open area (average and maximum particle sizes) and the applied force were analyzed and discussed further.

## 2. Materials and Methods

A commercial Novbelt conveyor belt material, obtained from Konus-Konex d.o.o. (Slovenia), was used initially to develop an auxetic textile material. The idea was to create a multifunctional material, e.g., a material used as a conveyor belt, with a filtration function capable of filtering particles of different sizes, depending on the load applied to the tension of the conveyor belt. Novbelt is a compound fabric made from three layers: The upper and lower layers are nonwoven PET fibrous webs, made by a conventional carding system and bonded with needle-punching technology; the middle, e.g., reinforcement layer, is plain-woven fabric, made from 111 tex PET filaments, with a thread density of 6/2 ends/picks per cm. Both types of compound fabrics’ components (woven and non-woven fabric) have a positive Poisson’s ratio as well as final compound fabric. The Poison’s ratio was not measured while from the theory it is well known that such materials don’t possess auxetic behaviour. The constructional parameters of the Novbelt compound fabric are listed in Table 1. 

**Figure 1:**
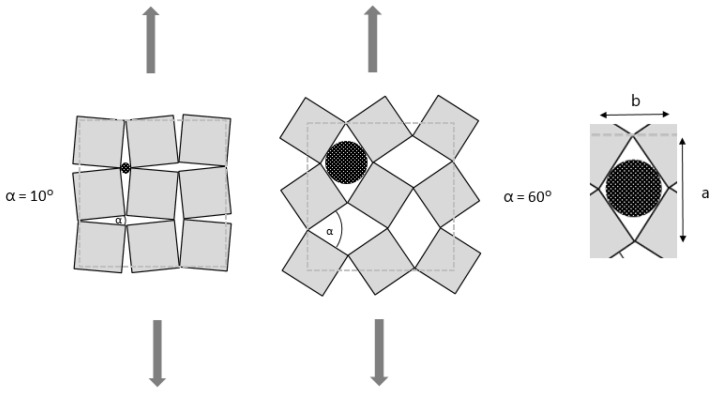
Deformation of fabric with rotating square unit cells geometry under loading (dashed lines–the initial size of the specimen) with particles of different sizes going through the fabric’s open area depending on the applied tension force (a and b are rhombus diagonals).

The deformation mechanism is shown schematically in Figure 1 and is grounded on a (quasi)-rigid squares arrangement connected at their vertices by hinges. The geometry of rotating square unit cells was induced by laser cutting (Epilog Helix 24 with 50 watts laser) to transform the conventional textile material (e.g., compound fabric) into an auxetic one. The principle of deformation of rotating unit cells under loading is shown in Figure 1.

Three rotating square unit cell sizes were studied in this research, i.e., 12.5 mm × 12.5 mm, 6.25 mm × 6.25 mm, and 3.125 mm × 3.125 mm. The hinge sizes and slit thicknesses remained the same for all tested samples. The details of the applied rotating square unit cell geometry are given in Table 2. The geometry of rotating cells refers to a network of rigid squares. One repeat unit of rotating cells is formed of four squares connected with the 2 mm long hinges at their corners with neighbourhood squares. Slit thicknesses between the rotating squares was 0.2 mm. Applied tensile force in the longitudinal direction causes these squares to rotate around hinges relative to each other, and consequently, the open pores within the structure are formed. The material expands in the lateral direction, and hence auxetic behavior is achieved. 

The Tinius Olsen H10KT tensile loading machine with 1 kN (auxetic samples) and 10 kN (original samples) load cells was used to measure the force required to apply a uniaxial tensile strain at a constant rate of extension, following the ISO EN 9073 Standard. Five samples with the dimensions of 250 mm ± 0.5 mm × 50 mm ± 0.5 mm (length × width) were prepared in the machine (MD) and cross-machine (CMD) directions for each type of specimen, and then subjected to conditioning for 48 h. Tensile measurements were conducted under the following testing conditions: The constant rate of the extension was 100 mm/min, the distance between the clamps was 150 mm, testing was carried out under standard climatic conditions. The elongation at break and maximum breaking force were calculated as an average of five measurements for all samples, expressed in N and %, respectively.

The deformation under quasi-static tensile measurements was first captured by a high-resolution camera, and later analyzed using a video image recognition methodology and a developed video analysis software (Accord.NET) to determine and analyze the Poisson’s ratio of all tested samples. The Poisson’s ratio was determined from the changes in the width of the samples versus the changes in the height of the samples, which was measured over time. The changes in the height of the specimen and the area of the video where the sample is located were determined based on the position of the moving clamp of the tensile machine. This position was determined using a matching object tracker for each video image (frame) and thus enabled the determination of the sample’s height over time. Then, image filtering was performed by using the Canny edge detector. Lastly, from the segmented image, the sample width was detected in pixels as the distance between two vertical lines on both sides of the sample. The side lines positions were determined in such a way that each line was moved from the border of the image towards the image center until it touched the sample in at least five distinct points. From the measured sample width change and height change over time, the time-dependent Poisson’s ratio was computed and compared, to evaluate the behavior of the developed auxetic compound fabrics.

The additional image video processing method was applied to analyze the influence of the tensile loading on the fabrics’ open area. As for the Poisson’s ratio determination, the images were segmented to a black and white image, where the background was depicted with white color. In this way, the fabric’s open areas were represented as white shapes on a black background. Then, the size was measured of the openings (pores) in the fabric. A spherical shape of the particles was assumed, to determine the particle size that would fit through the fabric’s opening. The longitudinal deformation of the rotating unit square structures results in rhombus openings between the squares. Thus, a rhombus inscribed circle radius determines the largest spherical particle that can pass through an opening. The inscribed circle radius was computed from both rhombus diagonals (Figure 1a,b), which were measured in pixels from the black and white images for all openings. The maximum particle size and the average particle size were computed for each time frame. It is important to emphasize that the average particle size, in this case, represents the average size of the largest particles that can fit through the fabric open areas.

In the case when the material is used as a filter media, the bursting strength is also an important factor to judge the durability of the material for filtration function. At this stage, the presented research is focused only on the material’s tensile strength to assess if compound material possesses auxetic behavior and how big particles can be filtered depending on the applied tensile load.

## 3. Results and Discussion

### 3.1. Tensile Behaviour Analysis

**Figure 2:**
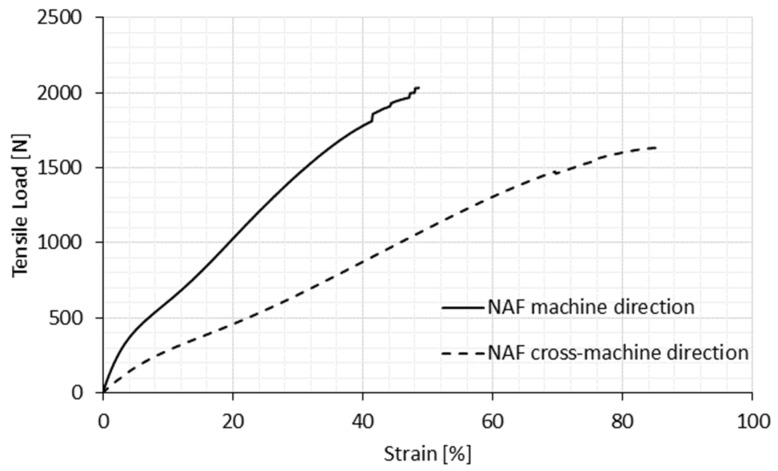
Tensile behavior of non-auxetic compound fabric (longitudinal deformation).

**Figure 3:**
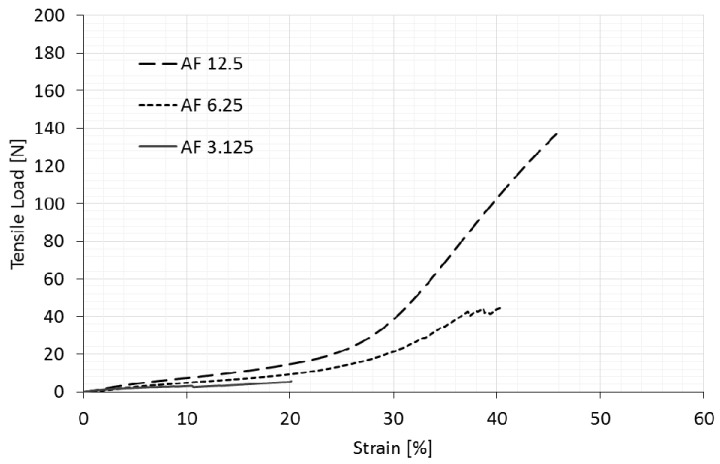
Tensile behavior of auxetic compound fabrics in the machine direction (longitudinal deformation).

**Figure 4:**
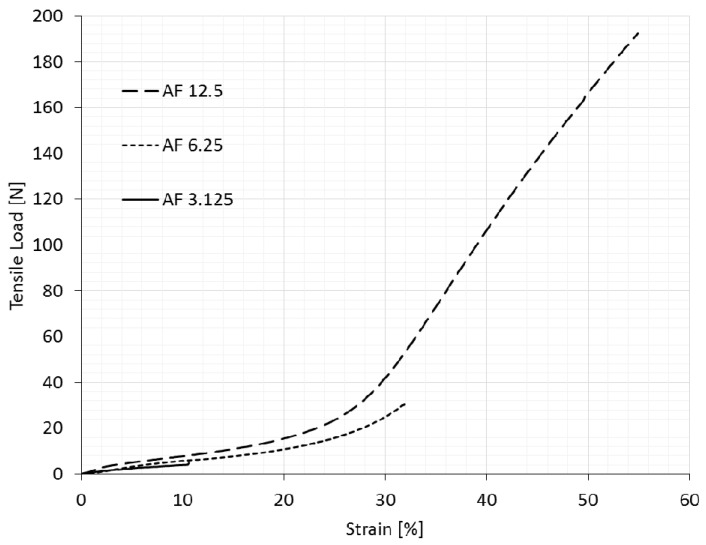
Tensile behavior of auxetic compound fabrics in the cross-machine direction (longitudinal deformation).

**Figure 5:**
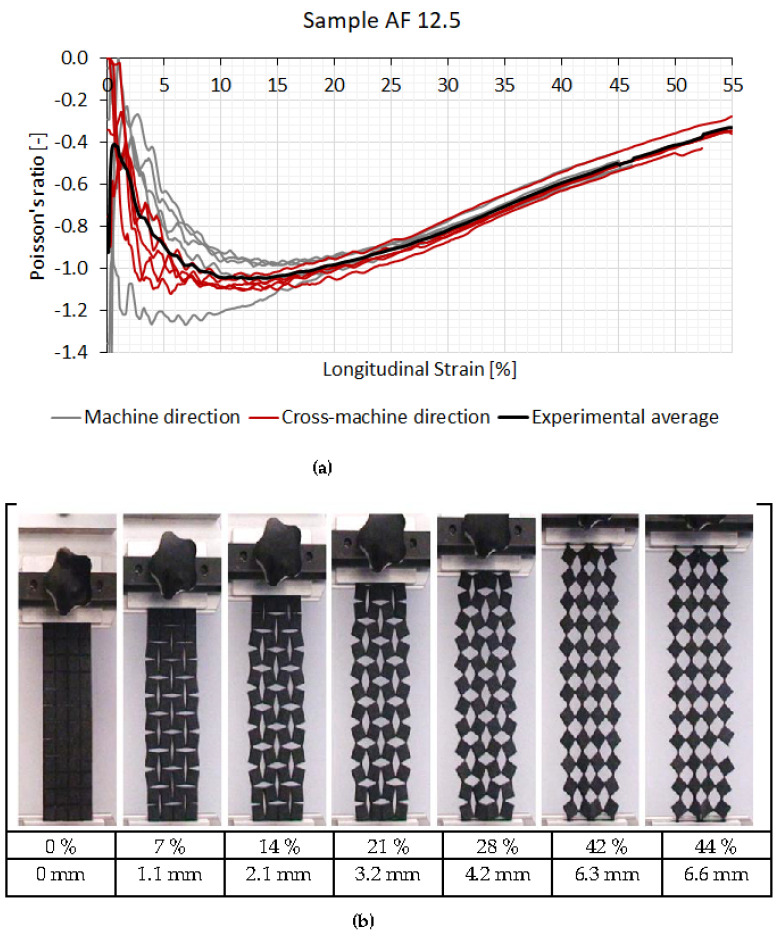
Poisson’s ratio of the AF 12.5 sample with the longitudinal strain (**a**) and corresponding fabric deformation in the machine-direction (**b**) (LS–longitudinal strain (%), LD–longitudinal displacement (mm)).

**Figure 6:**
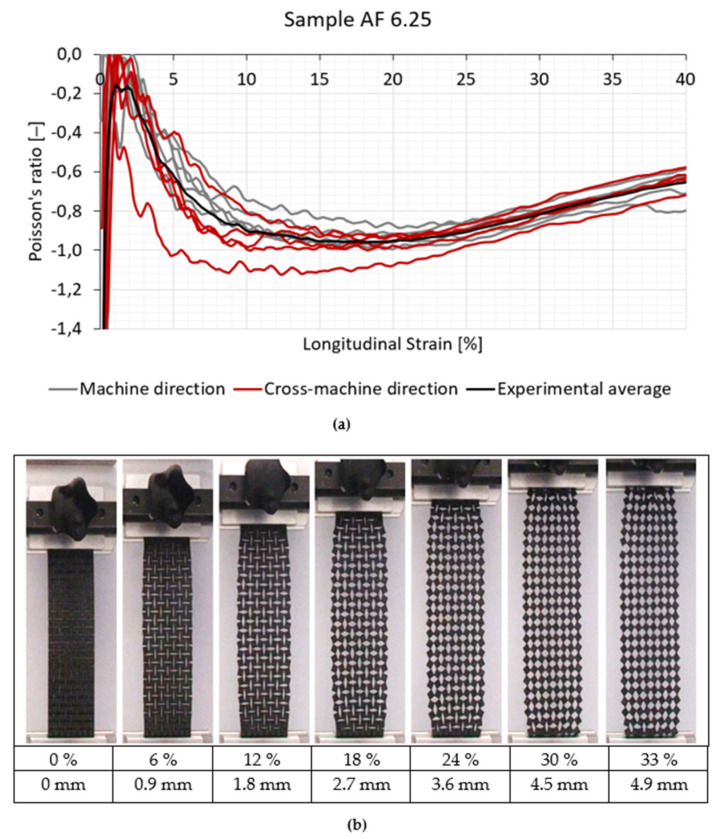
Poisson’s ratio of the AF 6.25 sample with the longitudinal strain (**a**) and corresponding fabric deformation in the machine-direction (**b**) (LS–longitudinal strain (%), LD–longitudinal displacement (mm)).

The longitudinal deformation of the original (non-auxetic) compound fabric (NAF) under tensile load in both machine and cross-machine directions is shown in Figure 2. Figure 3 and Figure 4 show auxetic compound fabrics (AF) longitudinal deformation under tensile load for three different rotating unit cell sizes (AF 12.5, AF 6.25, and AF 3.125) in the machine and cross-machine directions, respectively. The reported strains in Figure 2, Figure 3, Figure 4, Figure 5 and Figure 6 are the engineering strains based on the deformation of the sample as a whole. Table 3 shows the tensile properties measurements for non-auxetic (NAF) and auxetic compound fabrics (AF) with a coefficient of variation. 

From Figure 2 and Table 3, it can be observed that the original compound fabric has higher breaking strength in the machine direction, lower elongation at break and offers higher stiffness due to its structure. If we assume that nonwoven layers have equal fiber orientation in both directions, then the reinforcement layer, e.g., the plain-woven fabric, plays an essential role in tensile behavior. While a plain-woven fabric has a higher thread density in the warp direction (6 ends/cm), which is also the direction of loading, more threads can oppose the tensile load applied to the fabric in this direction than in the cross-machine (weft) direction (where the thread density is only 2 picks/cm). It is also possible that nonwoven layers have better fiber orientation in the machine direction, thus, additionally improving the resistance of fabric against deformation in this direction, and the difference of breaking strength and elongation is higher in comparison with the opposite direction.

Comparing the behavior of three different auxetic geometries under tensile load (Figure 3 and Figure 4) shows that larger unit cell size samples exhibit a higher breaking strength and elongation. This comparison was valid for samples in the machine and cross-machine directions. The result was expected, as samples with a smaller rotating unit cell size have more cuts per fabric unit area, which, consequently, destabilizes the structure of the material to resist the load. It should be noted how substantial the difference was between the geometries in relation to the resistance to tensile load. Results show that the AF 6.25 sample, in comparison with the AF 12.5 sample, had a 68.3% and 64.2% reduction of breaking strength in the machine and cross-machine directions, respectively. In contrast, the AF 3.125 sample shows a 95.9% and 97.4% reduction of breaking strength, limiting its usefulness for multifunctional applications, and was omitted in further discussion.

The results also show that the original (non-auxetic) sample possesses a higher breaking strength in the machine direction. In contrast, auxetic samples with 12.5 mm and 6.25 mm unit cell size demonstrated the opposite behavior (the breaking strength in the cross-machine direction was higher). However, the cutting pattern was the same in both directions. This can be explained by greater laser cutting damage to the reinforcement layer (woven fabric) of the compound fabric in the longitudinal (machine) direction, with more warp threads per cm than in the weft (cross-machine) direction.

The original (non-auxetic) and auxetic samples have a higher breaking elongation in the cross-machine direction, which is a consequence of the reinforcement layer having a higher crimp in the weft (cross-machine) direction than in the warp (machine) direction. The difference is evident in the original fabric (the 75% higher breaking elongation in the CD direction), while rotating square geometries reduced this difference (the elongation at break in the cross-machine direction was, on average, only 10% higher). By inducing auxetic geometry in the compound fabric, the inner structure of the compound fabric is affected–the threads in the reinforcement layer are cut, and there is no possibility to extend them. Furthermore, the induced squares start to rotate under the tension force, thus reducing the longitudinal extension.

The induced auxetic geometry in compound fabric influences the initial breaking force of the conventional textile material drastically. The average reduction of breaking force for both directions (without considering the AF 3.125 samples) was 94%. Therefore, the developed auxetic material can be used in cases where lower tensile strength suffices.

### 3.2. Poisson’s Ratio Analysis

Figure 5 and Figure 6 represent the results of the Poisson’s ratio of the tested auxetic specimens and resultant fabric deformations for AF 12.5 and AF 6.25 specimens, respectively. The deformation of both analysed auxetic samples (AF 12.5 and AF 6.25) was the same as predicted theoretically, i.e., the rotation of the quasi-rigid squares around their vertices (Figure 1). At strains above 30% the out-of-plane deformation appeared, due to the large deformation of the hinges, and resulted in a stiffness increase (Figure 3 and Figure 4).

All samples showed the negative Poison’s ratio already at low strains. After the longitudinal strain was applied, the unit cells started to rotate around the uncut parts–hinges. The width of the sample increased, and in-plane auxetic behavior was detected. The lowest average Poisson’s ratio of the tested samples was −1.05 at 13.9% strain for the AF 12.5 sample and −0.96 at 18.2% strain for the AF 6.25 sample. The NPR (negative Poisson’s ratio) existed within the entire longitudinal strain range for both geometries. Beyond the 30% of longitudinal strain, the specimens with smaller rotating unit cells exhibited a higher NPR (−0.65) until the rupture than the samples with a larger rotating unit cell size, where the Poisson’s ratio was −0.33.

The results show that compound fabrics with larger rotating unit cell size exhibit a higher average NPR. This is contrary to the similar research already done by the authors (Dubrovski et al., 2019) but on another textile material, e.g., needle-punched nonwoven fabrics, where fabrics with smaller rotating unit cell size achieved a higher average NPR. Such results are the consequence of the type of tested fabrics, while the geometry of the induced rotating cells was the same. The nonwoven fabric in the previous research was a much thinner (0.7 mm) and flexible textile material with a lower breaking strength (217–459 N/5 cm) in comparison with the compound fabric involved in this research, which was a thick (3.0 mm) and stiff textile material with a higher breaking strength (1628.3–2030 N/5 cm). In the case of more flexible material with larger rotating cells, the out-of-plane rotation occurs already at lower longitudinal strain, which reduces the extension in the lateral direction. Consequently, a lower average NPR is achieved compared to the flexible fabrics with smaller rotating cells, where only in-plane rotation was detected. In the case of a stiff textile material, such as a compound fabric, the out-of-plane rotation also occurred in the case of fabrics with larger rotating cells, but at a higher longitudinal strain. In the region of lower longitudinal strain only in-plane rotation of cells was detected, and higher average NPR was achieved compared to the fabrics with a smaller unit cell size. However, beyond the 30% of longitudinal strain, the compound fabric with larger rotating unit cells, because of the out-of-plane rotation, again exhibited a lower NPR than the compound fabrics with a smaller rotating unit cell.

From Figure 5a, with applied longitudinal strain, the Poisson’s ratio varied from −1.0 to −0.4. However, the difference between the machine and the cross-machine direction was minor. It was related to the rotation of the unit cells, or added auxetic geometry (which, consequently, reduces the Poisson’s ratio effect), and not to the original fabric structure, which does not exhibit auxetic properties. In this research (Figure 5a), some samples show the Poisson’s ratio lower than −1 due to the initial alignment of the unit cells at small deformations, where the transverse deformation behavior is not representative.

The Poisson’s ratio relationship to longitudinal strain is not smooth. Many local extremes occur from 0% to approximately 30% of the longitudinal strain for both geometries. The extremes correspond to the rotation of the squares. At the start, the rotation of squares is higher in the sample center than on its boundaries. After the maximum Poisson’s ratio is detected, the rotating of squares continues, mostly in the area away from the sample center, until rotation stops (no more extremes are identified) and curves smoothen. In this region, the material properties, especially at the hinges, are crucial to withstand the tensile loading.

### 3.3. Fabric’s Open Area and Particle Size Analysis

**Figure 7:**
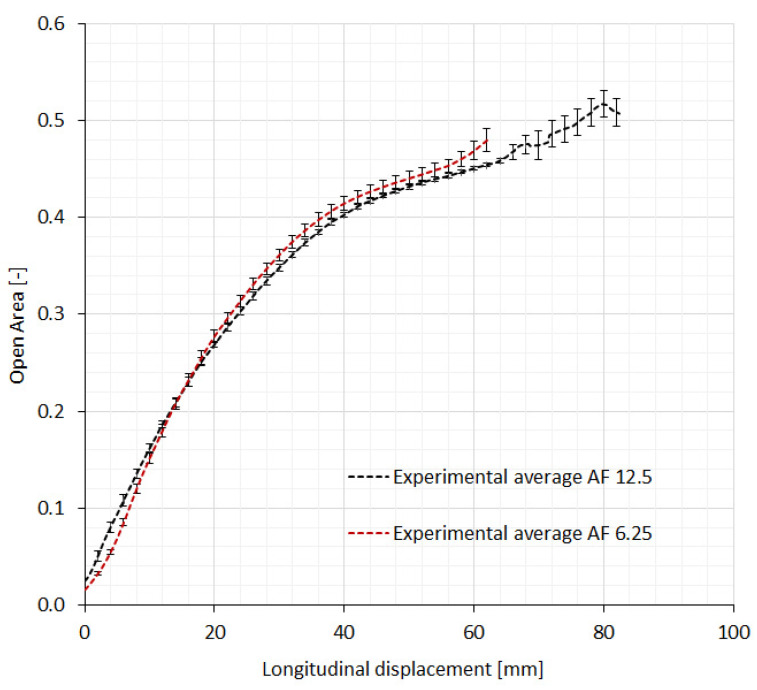
The relationship between the open area and longitudinal displacement.

Figure 7 shows the relationship between the fabric’s open area and longitudinal displacement during the tensile testing for both rotating square unit geometries. Here, the fabric’s open area (the ratio between the area of pores and the fabric area unit) is expressed as a factor. The relationships represent the average values of all tested samples in the machine and cross-machine directions. The measurement uncertainty is also added to the average values. At the start of the tensile testing, there is approximately 0.2 of the open area due to the 0.2 mm cuts. The square cells start to rotate with increasing longitudinal displacement, thus forming voids in the shape of rhombi, and increasing the fabric’s open area almost linearly up to approximately 0.28, and then with a slowly decreasing rate until breakage at around 0.5. There was a slight difference between both geometries. The smaller unit cell size samples have a higher open area at displacements larger than 15 mm. The results show that the maximum open areas at break equal 0.48 and 0.51 for AF 12.5 and AF 6.25 samples, respectively. 

**Figure 8:**
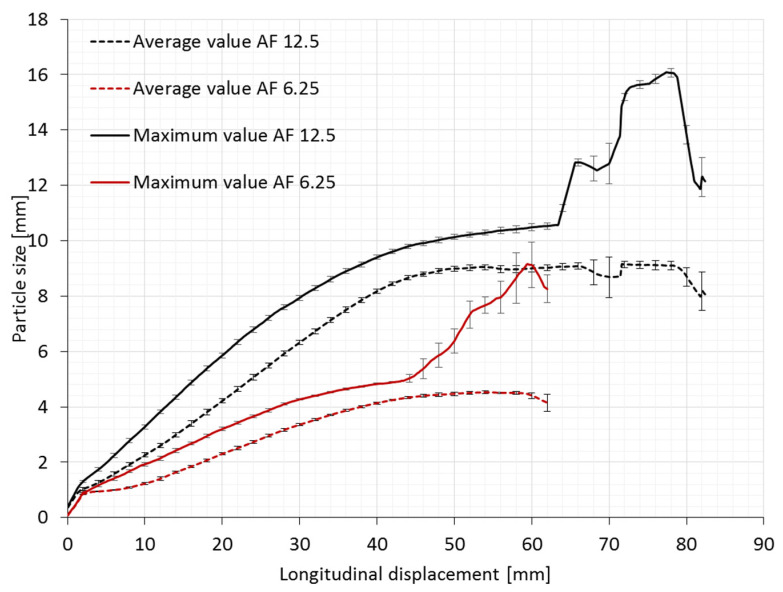
The relationship between the particle sizes and longitudinal displacement.

For application, the information about the largest particle that can fit through the open area is more valuable (see detail in Figure 1). This information relates the longitudinal displacement to the largest particle size which can fit through the open area, and determines the desired particle size for the filtration process. Figure 8 represents the dependence between the particle sizes and the longitudinal displacement of the tested fabrics for both geometries, regardless of the direction of applied tension. 

The results show clearly that a 12.5 mm rotating unit cell size auxetic compound fabric can filter particles with the average size ranging from 1 mm to 9 mm, depending on the tensile load applied to the conveyor belt. In the auxetic compound fabric with the rotating unit cell size of 6.25 mm, particles from 1 mm to 4.5 mm can be filtered before failure. 

Open areas of different sizes are formed during tensile loading. The size dependence of the largest particle that can fit through the open areas is presented in Figure 8. It is observed that the maximum particle size of 10.5 mm can pass through the filter at approximately 64 mm of longitudinal displacement (approximately 43% of longitudinal strain) by auxetic samples with larger unit cell size. In comparison, in the case of auxetic samples with smaller unit cell size, the maximum particle size is lower. It reaches 5 mm at 44.4 mm of longitudinal displacement (approximately 30% of longitudinal strain). Both relationships of maximum particle sizes show a much higher increase from the previous mentioned longitudinal displacement (the last third of the maximum particle size’s curves). This corresponds to the situation where an extensive failure of the fabric’s structure occurs when hinges start to break (Figure 5b and Figure 6b). At the same time, the geometry of voids (rhombi) starts to break at particular positions, where hinges are interrupted, until the final fabric’s break.

## 4. Conclusions

In this work, a multifunctional (transportation, filtration) compound fabric with auxetic geometry was designed using a conventional textile material (compound fabric) with laser-cut slits in the form of a symmetric auxetic pattern (rotating squares). The deformation behavior and Poisson’s ratio of the developed fabrics were evaluated using digital imaging and analysis. The rotation of the squares under tensile loading forms an open area, which controls the size of the particles that can pass through (filtration). The fabric’s open area and average particle size were determined in relation to the longitudinal displacement. The developed material can be used as a filter media with a variable open area for filtering different sizes of particles, depending on the applied auxetic geometry and tensile load. The conclusions of this study can be summarized as follows:

- A conventional textile material can be transformed into a multifunctional one by laser cutting of the predefined geometry of the rotating squares. The developed auxetic compound fabrics exhibited Poisson’s ratios ranging from 0 to values lower than –1.
- Fabrics with induced larger unit cells exhibited a higher average negative Poisson’s ratio. The lowest experimental average Poisson’s ratio of tested samples was −1.05 at 13.9% of strain for auxetic samples with a unit cell size of 12.5 mm, and −0.96 at 18.2% of strain for auxetic fabrics with a unit cell size of 6.25 mm. However, beyond 30% of longitudinal strain, the smaller unit cell size specimens showed a higher NPR across the whole longitudinal strain range until failure.
- The introduction of auxetic geometry into compound fabric leads to a major tensile strength reduction. Fabrics with larger unit cell size show a higher breaking force and elongation under tensile load. Their application is, thus, restricted to low tensile loads.
- Fabrics with larger unit cell size offer a higher particle size range for particle filtering, depending on the applied tension load.

Further research will be focused on the parametric study of geometric parameters and their influence on mechanical and deformation behavior. Different patterns of slits or rotating unit structures into the conventional textile material will also be considered. The reduction of tensile strength will be minimized by cutting the initial textile material.

## Figures and Tables

**Table 1:** The constructional parameters of the Novbelt compound fabric.

Sample ID	Raw Material	Fabric Mass per Unit Area (gm^−2^)	Fabric Thickness (mm)	Fabric Density (gcm^−3^)
CF	PET	1292	2.978	0.434

**Table 2:** The geometry of the laser cutting pattern and rotating cells.

One Repeat Unit of Laser Cutting Pattern (4 cells)	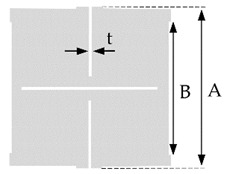		
Size of one cell (mm)	12.5	6.25	3.125
A (mm)	25	12.5	6.25
B (mm)	23	10.5	4.25
t (mm)	0.2	0.2	0.2
Sample Code	AF 12.5	AF 6.25	AF 3.125

**Table 3:** Tensile properties‘ measurements (CV (%)–coefficient of variation).

		Breaking Force (N)		Breaking Elongation (%)	
Sample Code		Machine Direction	Cross-Machine Direction	Machine Direction	Cross-Machine Direction
NAF		2030	1628.3	48.5	85.1
	CV	8.1	4.6	6.6	8.9
AF 12.5		140.5	192.3	46.3	54.9
	CV	7.6	10.1	4.3	6.7
AF 6.25		44.6	65.2	40.4	41.1
	CV	14.4	9.7	3.6	1.6
AF 3.125		5.7	4.8	20.1	10.6
	CV	42.3	48.7	42.8	24.1

## Data Availability

The data that support the findings of this study are available from the corresponding author upon reasonable request.
